# On Hardy-type integral inequalities with the gamma function

**DOI:** 10.1186/s13660-017-1404-1

**Published:** 2017-06-07

**Authors:** Jianquan Liao, Bicheng Yang

**Affiliations:** Department of Mathematics, Guangdong University of Education, Guangzhou, Guangdong 51003 P.R. China

**Keywords:** 26D15, Hardy-type integral inequality, weight function, equivalent form, operator, norm

## Abstract

By means of real analysis and weight functions, we obtain a few equivalent conditions of two kinds of Hardy-type integral inequalities with the non-homogeneous kernel and parameters. The constant factors related to the gamma function are proved to be the best possible. We also consider the operator expressions and some cases of homogeneous kernel.

## Introduction

If $0 < \int_{0}^{\infty } f^{2}(x)\,dx < \infty $ and $0 < \int_{0} ^{\infty } g^{2}(y)\,dy < \infty $, then we have the following Hilbert’s integral inequality (*cf.* [1]):

1 $$\begin{aligned}& \int_{0}^{\infty } \int_{0}^{\infty } \frac{f(x)g(y)}{x + y}\,dx\,dy< \pi \biggl( \int_{0}^{\infty } f^{2}(x)\,dx \int_{0}^{\infty } g^{2}(y)\,dy \biggr) ^{\frac{1}{2}}, \end{aligned}$$

where the constant factor *π* is the best possible. In 1925, by introducing one pair of conjugate exponents (*p*, *q*), Hardy [2] gave an extension of (1) as follows: For $p > 1$, $\frac{1}{p} + \frac{1}{q} = 1$, $f(x)$, $g(y) \ge 0$, $0 < \int_{0}^{\infty } f^{p}(x)\,dx < \infty$ and $0 < \int_{0}^{\infty } g^{q}(y)\,dy < \infty$, we have

2 $$\begin{aligned}& \int_{0}^{\infty } \int_{0}^{\infty } \frac{f(x)g(y)}{x + y}\,dx\,dy < \frac{ \pi }{\sin (\pi / p)} \biggl( \int_{0}^{\infty } f^{p}(x)\,dx \biggr) ^{ \frac{1}{p}} \biggl( \int_{0}^{\infty } g^{q}(y)\,dy \biggr) ^{\frac{1}{q}}, \end{aligned}$$

where the constant factor $\frac{\pi }{\sin (\pi / p)}$ is the best possible. Inequalities (1) and (2) are important in analysis and its applications (*cf.* [3, 4]).

In 1934, Hardy *et al*. gave an extension of (2) as follows: If $k_{1}(x,y)$ is a non-negative homogeneous function of degree −1, $k_{p} = \int_{0}^{\infty } k_{1}(u,1)u^{\frac{ - 1}{p}}\,du \in \mathbf{R}_{ +} = (0,\infty)$, then

3 $$\begin{aligned}& \int_{0}^{\infty } \int_{0}^{\infty } k_{1}(x,y)f(x)g(y)\,dx\,dy < k_{p} \biggl( \int_{0}^{\infty } f^{p}(x)\,dx \biggr) ^{\frac{1}{p}} \biggl( \int _{0}^{\infty } g^{q}(y)\,dy \biggr) ^{\frac{1}{q}}, \end{aligned}$$

where the constant factor $k_{p}$ is the best possible (*cf.* [3], Theorem 319). Additionally, a Hilbert-type integral inequality with the non-homogeneous kernel is proved as follows: If $h(u) > 0$, $\phi (\sigma) = \int_{0}^{\infty } h(u)u^{\sigma - 1}\,du \in \mathbf{R}_{ +}$, then

4 $$\begin{aligned}& \int_{0}^{\infty } \int_{0}^{\infty } h(xy)f(x)g(y)\,dx\,dy \\& \quad < \phi \biggl(\frac{1}{p} \biggr) \biggl( \int_{0}^{\infty } x^{p - 2}f^{p}(x)\,dx \biggr) ^{\frac{1}{p}} \biggl( \int_{0}^{\infty } g^{q}(y)\,dy \biggr) ^{ \frac{1}{q}}, \end{aligned}$$

where the constant factor $\phi (\frac{1}{p})$ is still the best possible (*cf.* [3], Theorem 350).

In 1998, by introducing an independent parameter $\lambda > 0$, Yang gave a best extension of (1) with the kernel $\frac{1}{ ( x + y )^{\lambda }}$ (*cf.* [5, 6]). In 2004, by introducing another pair conjugate exponents (*r*, *s*), Yang [7] gave an extension of (2) as follows: If $\lambda > 0$, $r > 1,\frac{1}{r} + \frac{1}{s} = 1$, $f(x)$, $g(y) \ge 0$, $0 < \int_{0}^{\infty } x^{p ( 1 - \frac{\lambda }{r} ) - 1}f^{p}(x)\,dx < \infty $ and $0 < \int_{0}^{\infty } y^{q (1 -\frac{\lambda }{s} ) - 1}g^{q}(y)\,dy < \infty $, then

5 $$\begin{aligned}& \int_{0}^{\infty } \int_{0}^{\infty } \frac{f(x)g(y)}{x^{\lambda } + y ^{\lambda }} \,dx\,dy \\& \quad < \frac{\pi }{\lambda \sin ( \pi / r ) } \biggl( \int_{0}^{ \infty } x^{p ( 1 - \frac{\lambda }{r} ) - 1}f^{p}(x) \,dx \biggr) ^{\frac{1}{p}} \biggl( \int_{0}^{\infty } y^{q ( 1 - \frac{\lambda }{s} ) - 1}g^{q}(y)\,dy \biggr) ^{\frac{1}{q}}, \end{aligned}$$

where the constant factor $\frac{\pi }{\lambda \sin ( \pi / r ) }$ is the best possible. For $\lambda = 0$, $r = q$, $s = p$, (5) reduces to (2); For $\lambda = 1$, $r = p$, $s = q$, (5) reduces to the dual form of (2) as follows:

6 $$\begin{aligned}& \int_{0}^{\infty } \int_{0}^{\infty } \frac{f(x)g(y)}{x + y}\,dx\,dy \\& \quad < \frac{\pi }{\sin ( \pi / p ) } \biggl( \int_{0}^{\infty } x ^{p - 2}f^{p}(x) \,dx \biggr) ^{\frac{1}{p}} \biggl( \int_{0}^{\infty } y ^{q - 2}g^{q}(y) \,dy \biggr) ^{\frac{1}{q}}. \end{aligned}$$

For $p = q = 2$, both (2) and (7) reduce to (1).

In 2005, in [8] one also gave an extension of (2) and (5) with the kernel $\frac{1}{ ( x + y ) ^{\lambda }}$. Krnić *et al*. [9–14] provided some extensions and particular cases of (2), (3) and (4) with parameters.

In 2009, Yang gave an extension of (3) and (5) as follows (*cf.* [15, 16]): If $\lambda_{1} + \lambda_{2} = \lambda \in \mathbf{R} = (- \infty ,\infty)$, $k_{\lambda } (x,y)$ is a non-negative homogeneous function of degree −*λ*, satisfying

$$ k_{\lambda } (ux,uy) = u^{ - \lambda } k_{\lambda } (x,y) \quad (u,x,y > 0), $$

and

$$ k(\lambda_{1}) = \int_{0}^{\infty } k_{\lambda } (u,1)u^{\lambda_{1} - 1} \,du \in \mathbf{R}_{ +}, $$

then

7 $$\begin{aligned}& \int_{0}^{\infty } \int_{0}^{\infty } k_{\lambda } (x,y)f(x)g(y)\,dx\,dy \\& \quad < k(\lambda_{1}) \biggl( \int_{0}^{\infty } x^{p ( 1 - \lambda_{1} ) - 1}f^{p}(x)\,dx \biggr) ^{\frac{1}{p}} \biggl( \int_{0}^{\infty } y^{q ( 1 - \lambda_{2} ) - 1}g^{q}(y)\,dy \biggr) ^{ \frac{1}{q}}, \end{aligned}$$

where the constant factor $k(\lambda_{1})$ is the best possible. For $\lambda = 1$, $\lambda_{1} = \frac{1}{q}$, $\lambda_{2} = \frac{1}{p}$, (7) reduces to (3). Additionally, an extension of (4) was given as follows:

8 $$\begin{aligned}& \int_{0}^{\infty } \int_{0}^{\infty } h(xy)f(x)g(y)\,dx\,dy \\& \quad < \phi (\sigma) \biggl( \int_{0}^{\infty } x^{p ( 1 - \sigma ) -1}f^{p}(x)\,dx \biggr) ^{\frac{1}{p}} \biggl( \int_{0} ^{\infty } y^{q ( 1 -\sigma ) - 1}g^{q}(y) \,dy \biggr) ^{\frac{1}{q}}, \end{aligned}$$

where the constant factor $\phi (\sigma)$ is the best possible (*cf.* [17]). For $\sigma = \frac{1}{p}$, (8) reduces to (4).

Some equivalent inequalities of (7) and (8) are considered by [16]. In 2013, Yang [17] studied the equivalency between (7) and (8). In 2017, Hong [18] studied an equivalent condition between (7) with a few parameters.

### Remark 1

*cf.* [17]

If $h(xy) = 0$, for $xy > 1$, $\phi (\sigma) = \int_{0}^{1} h(u)u^{\sigma - 1}\,du = \phi_{1}(\sigma) \in \mathbf{R}_{ +}$, then (8) reduces to the following Hardy-type integral inequality with the non-homogeneous kernel:

9 $$\begin{aligned}& \int_{0}^{\infty } g(y) \biggl( \int_{0}^{\frac{1}{y}} h(xy)f(x)\,dx \biggr)\,dy \\& \quad < \phi_{1}(\sigma) \biggl( \int_{0}^{\infty } x^{p ( 1 - \sigma ) - 1}f^{p}(x)\,dx \biggr) ^{\frac{1}{p}} \biggl( \int_{0}^{\infty } y^{q ( 1 - \sigma ) - 1}g^{q}(y)\,dy \biggr) ^{\frac{1}{q}}; \end{aligned}$$

if $h(xy) = 0$, for $xy < 1$, $\phi (\sigma) = \int_{1}^{\infty } h(u)u ^{\sigma - 1}\,du = \phi_{2}(\sigma) \in \mathbf{R}_{ +}$, then (8) reduces to the following another kind of Hardy-type integral inequality with the non-homogeneous kernel:

10 $$\begin{aligned}& \int_{0}^{\infty } g(y) \biggl( \int_{\frac{1}{y}}^{\infty } h(xy)f(x)\,dx \biggr)\,dy \\& \quad < \phi_{2}(\sigma) \biggl( \int_{0}^{\infty } x^{p ( 1 - \sigma ) - 1}f^{p}(x)\,dx \biggr) ^{\frac{1}{p}} \biggl( \int_{0}^{\infty } y^{q ( 1 - \sigma ) - 1}g^{q}(y)\,dy \biggr) ^{\frac{1}{q}}. \end{aligned}$$

In this paper, by real analysis and the weight functions, we obtain a few equivalent conditions of two kinds of Hardy-type integral inequalities with the non-homogeneous kernel and parameters as $\frac{ ( \min \{ xy,1 \} ) ^{\alpha } \vert \ln xy \vert ^{\beta }}{ ( \max \{ xy,1 \} ) ^{\lambda + \alpha }}$. The constant factors related to the gamma function are proved to be the best possible. We also consider the operator expressions and some cases of homogeneous kernel.

## Two lemmas

For $\beta > - 1$, $\sigma + \mu = \lambda \in \mathbf{R}$, we set

$$\begin{aligned}& h(u): = \frac{( \min \{ u,1 \}) ^{\alpha } \vert \ln u \vert ^{\beta }}{ ( \max \{ u,1 \} ) ^{\lambda + \alpha}}\quad (u > 0). \end{aligned}$$

Then, for $\sigma > - \alpha $, setting $v = - (\sigma + \alpha)\ln u$, we find

11 $$\begin{aligned} k_{1}(\sigma)&: = \int_{0}^{1} \frac{ ( \min \{ u,1 \} ) ^{\alpha } \vert \ln u \vert ^{\beta }}{ ( \max \{ u,1 \} ) ^{\lambda + \alpha }} u^{\sigma - 1} \,du \\ &= \int_{0}^{1} u^{\sigma + \alpha - 1} ( - \ln u ) ^{\beta }\,du = \frac{1}{ ( \sigma + \alpha ) ^{\beta + 1}} \int_{0}^{ \infty } v^{\beta } e^{ - v}\,dv= \frac{\Gamma ( \beta + 1 ) }{ ( \sigma + \alpha ) ^{\beta + 1}} \in \mathbf{R}_{ +}. \end{aligned}$$

For $\mu > - \alpha $, we find

12 $$\begin{aligned} k_{2}(\sigma)&: = \int_{1}^{\infty } \frac{ ( \min \{ u,1 \} ) ^{\alpha } \vert \ln u \vert ^{\beta }}{ (\max \{ u,1 \} ) ^{\lambda + \alpha }} u^{\sigma - 1}\,du \\ & = \int_{0}^{1} v^{\mu + \alpha - 1} ( - \ln v ) ^{\beta }\,dv = \frac{\Gamma ( \beta + 1 ) }{ ( \mu + \alpha ) ^{\beta + 1}} = k_{1}(\mu) \in \mathbf{R}_{ +}, \end{aligned}$$

where $\Gamma ( \eta ) : = \int_{0}^{\infty } v^{\eta - 1}e ^{ - v}\,dv$ ($\eta > 0$) is the gamma function (*cf.* [19]).

### Lemma 1

*If* $p > 1$, $\frac{1}{p} + \frac{1}{q} = 1$, $\sigma_{1} \in R$, $\beta > - 1$, $\sigma > - \alpha $, *there exists a constant* $M_{1}$, *such that*, *for any non*-*negative measurable functions* $f(x)$ *and* $g(y)$ *in* (0, ∞), *the following inequality*:

13 $$\begin{aligned} & \int_{0}^{\infty } g(y) \biggl[ \int_{0}^{\frac{1}{y}} \frac{ (\min \{ xy,1 \} ) ^{\alpha } \vert \ln xy \vert ^{\beta }}{ ( \max \{ xy,1 \} ) ^{\lambda + \alpha }} f(x)\,dx \biggr] \,dy \\ &\quad \le M_{1} \biggl[ \int_{0}^{\infty } x^{p ( 1 - \sigma ) - 1}f ^{p}(x) \,dx \biggr] ^{\frac{1}{p}} \biggl[ \int_{0}^{\infty } y^{q ( 1 - \sigma_{1} ) - 1}g^{q}(y)\,dy \biggr] ^{\frac{1}{q}} \end{aligned}$$

*holds true*, *then we have* $\sigma_{1} = \sigma $, *and then* $M_{1} \ge k_{1}(\sigma)$.

### Proof

If $\sigma_{1} > \sigma $, then, for $n \ge \frac{1}{ \sigma_{1} - \sigma}$ ($n \in \mathbf{N}$), we set the following functions:

$$\begin{aligned} f_{n}(x): = \textstyle\begin{cases} x^{\sigma + \frac{1}{pn} - 1},&0 < x \le 1, \\ 0,&x > 1, \end{cases}\displaystyle \qquad g_{n}(y): = \textstyle\begin{cases} 0,&0 < y < 1, \\ y^{\sigma_{1} - \frac{1}{qn} - 1},&y \ge 1, \end{cases}\displaystyle \end{aligned}$$

and find

$$\begin{aligned} J_{1}&: = \biggl[ \int_{0}^{\infty } x^{p ( 1 - \sigma ) - 1}f _{n}^{p}(x) \,dx \biggr] ^{\frac{1}{p}} \biggl[ \int_{0}^{\infty } y^{q ( 1 - \sigma_{1} ) - 1}g_{n}^{q}(y) \,dy \biggr] ^{\frac{1}{q}} \\ &= \biggl( \int_{0}^{1} x^{\frac{1}{n} - 1}\,dx \biggr) ^{\frac{1}{p}} \biggl( \int_{1}^{\infty } y^{ - \frac{1}{n} - 1}\,dy \biggr) ^{ \frac{1}{q}} = n. \end{aligned}$$

Setting $u = xy$, we obtain

$$\begin{aligned} I_{1}&: = \int_{0}^{\infty } g_{n}(y) \biggl( \int_{0}^{\frac{1}{y}} \frac{ ( \min \{ xy,1 \} ) ^{\alpha } \vert \ln xy \vert ^{\beta }}{ ( \max \{ xy,1 \} ) ^{\lambda + \alpha }} f_{n}(x)\,dx \biggr)\,dy \\ &= \int_{1}^{\infty } \biggl( \int_{0}^{\frac{1}{y}} \frac{ (\min \{ xy,1 \} ) ^{\alpha } ( - \ln xy ) ^{\beta }}{ (\max \{ xy,1 \} ) ^{\lambda + \alpha }} x^{\sigma + \frac{1}{pn} - 1} \,dx \biggr) y^{\sigma_{1} - \frac{1}{qn} - 1}\,dy \\ &= \int_{1}^{\infty } y^{ ( \sigma_{1} - \sigma ) - \frac{1}{n} - 1}\,dy \int_{0}^{1} \frac{ ( \min \{ u,1 \} ) ^{\alpha } ( - \ln u ) ^{\beta }}{ ( \max \{ u,1 \} ) ^{\lambda + \alpha }} u^{\sigma + \frac{1}{pn} - 1} \,du. \end{aligned}$$

Then by (13), we have

14 $$\begin{aligned}& \int_{1}^{\infty } y^{ ( \sigma_{1} - \sigma ) - \frac{1}{n} - 1}\,dy \int_{0}^{1} \frac{ ( \min \{ u,1 \} ) ^{\alpha } ( - \ln u ) ^{\beta }}{ ( \max \{ u,1 \} ) ^{\lambda + \alpha }} u^{\sigma + \frac{1}{pn} - 1} \,du \\& \quad = I_{1} \le M_{1}J_{1} = M_{1}n < \infty . \end{aligned}$$

Since $( \sigma_{1} - \sigma ) - \frac{1}{n} \ge 0$, it follows that $\int_{1}^{\infty } y^{ ( \sigma_{1} - \sigma ) - \frac{1}{n} - 1}\,dy = \infty $. By (14), in view of $\int_{0}^{ \frac{1}{y}} \frac{ ( \min \{ u,1 \} ) ^{\alpha } ( - \ln u ) ^{\beta }}{ ( \max \{ u,1 \} ) ^{\lambda + \alpha }} u^{\sigma + \frac{1}{pn} - 1}\,du > 0$, we find $\infty < \infty $, which is a contradiction.

If $\sigma_{1} < \sigma $, then, for $n \ge \frac{1}{\sigma - \sigma _{1}}$ ($n \in \mathbf{N}$), we set the following functions:

$$ \tilde{f}_{n}(x): = \textstyle\begin{cases} 0,&0 < x < 1, \\ x^{\sigma - \frac{1}{pn} - 1},&x \ge 1, \end{cases}\displaystyle \qquad \tilde{g}_{n}(y): = \textstyle\begin{cases} y^{\sigma_{1} + \frac{1}{qn} - 1}, &0 < y \le 1, \\ 0,&y > 1, \end{cases} $$

and find

$$\begin{aligned}& \tilde{J}_{1}: = \biggl[ \int_{0}^{\infty } x^{p ( 1 - \sigma ) - 1}\tilde{f}_{n}^{p}(x) \,dx \biggr] ^{\frac{1}{p}} \biggl[ \int_{0}^{ \infty } y^{q ( 1 - \sigma_{1} ) - 1}\tilde{g}_{n}^{q}(y) \,dy \biggr] ^{\frac{1}{q}} \\& \quad = \biggl( \int_{1}^{\infty } x^{ - \frac{1}{n} - 1}\,dx \biggr) ^{ \frac{1}{p}} \biggl( \int_{0}^{1} y^{\frac{1}{n} - 1}\,dy \biggr) ^{ \frac{1}{q}} = n. \end{aligned}$$

Setting $u = xy$, we obtain

$$\begin{aligned} \tilde{I}_{1}&: = \int_{0}^{\infty } \tilde{f}_{n}(x) \biggl( \int_{0} ^{\frac{1}{x}} \frac{ ( \min \{ xy,1 \} ) ^{ \alpha } \vert \ln xy \vert ^{\beta }}{ ( \max \{ xy,1 \} ) ^{\lambda + \alpha }} \tilde{g}_{n}(y)\,dy \biggr)\,dx \\ &= \int_{1}^{\infty } \biggl( \int_{0}^{\frac{1}{x}} \frac{ (\min \{ xy,1 \} ) ^{\alpha } ( - \ln xy ) ^{\beta }}{ (\max \{ xy,1 \} ) ^{\lambda + \alpha }} y^{\sigma_{1} + \frac{1}{qn} - 1} \,dy \biggr) x^{\sigma - \frac{1}{pn} - 1}\,dx \\ &= \int_{1}^{\infty } x^{ ( \sigma - \sigma_{1} ) - \frac{1}{n} - 1}\,dx \int_{0}^{1} \frac{ ( \min \{ u,1 \} ) ^{\alpha } ( - \ln u ) ^{\beta }}{ ( \max \{ u,1 \} ) ^{\lambda + \alpha }} u^{\sigma_{1} + \frac{1}{qn} - 1} \,du. \end{aligned}$$

Then by the Fubini theorem (*cf.* [20]) and (13), we have

15 $$\begin{aligned}& \int_{1}^{\infty } x^{ ( \sigma - \sigma_{1} ) - \frac{1}{n} - 1}\,dx \int_{0}^{1} \frac{ ( \min \{ u,1 \} ) ^{\alpha } ( - \ln u ) ^{\beta }}{ ( \max \{ u,1 \} ) ^{\lambda + \alpha }} u^{\sigma_{1} + \frac{1}{qn} - 1} \,du \\& \quad = \tilde{I}_{1} = \int_{0}^{\infty } \tilde{g}_{n}(y) \biggl( \int_{0} ^{\frac{1}{y}} \frac{ ( \min \{ xy,1 \} ) ^{ \alpha } \vert \ln xy \vert ^{\beta }}{ ( \max \{ xy,1 \} ) ^{\lambda + \alpha }} \tilde{f}_{n}(x)\,dx \biggr)\,dy \\& \quad \le M_{1}\tilde{J}_{1} = M_{1}n < \infty . \end{aligned}$$

Since $( \sigma_{1} - \sigma ) - \frac{1}{n} \ge 0$, it follows that $\int_{1}^{\infty } x^{ ( \sigma - \sigma_{1} ) - \frac{1}{n} - 1}\,dx = \infty $. By (15), in view of

$$ \int_{0}^{1} \frac{ ( \min \{ u,1 \} ) ^{\alpha } ( - \ln u ) ^{\beta }}{ ( \max \{ u,1 \} ) ^{\lambda + \alpha }} u^{\sigma_{1} + \frac{1}{qn} - 1} \,du > 0 $$

we find $\infty < \infty $, which is a contradiction.

Hence, we conclude that $\sigma_{1} = \sigma $.

For $\sigma_{1} = \sigma $, we reduce (15) as follows:

16 $$ M_{1} \ge \int_{0}^{1} \frac{ ( \min \{ u,1 \} ) ^{\alpha } ( - \ln u ) ^{\beta }}{ ( \max \{ u,1 \} ) ^{\lambda + \alpha }} u^{\sigma + \frac{1}{qn} - 1} \,du. $$

Since

$$ \biggl\{ \frac{ ( \min \{ u,1 \} ) ^{\alpha } ( - \ln u ) ^{\beta }}{ ( \max \{ u,1 \} ) ^{\lambda + \alpha }} u^{\sigma + \frac{1}{qn} - 1} \biggr\} _{n = 1} ^{\infty } $$

is non-negative and increasing in (0, 1], by Levi theorem (*cf.* [20]), we find

$$\begin{aligned} M_{1} &\ge \lim_{n \to \infty } \int_{0}^{1} \frac{ ( \min \{ u,1 \} ) ^{\alpha } ( - \ln u ) ^{\beta }}{ (\max \{ u,1 \} ) ^{\lambda + \alpha }} u^{\sigma + \frac{1}{qn} - 1} \,du \\ &= \int_{0}^{1} \lim_{n \to \infty } \frac{ ( \min \{ u,1 \} ) ^{\alpha } ( - \ln u ) ^{\beta }}{ (\max \{ u,1 \} ) ^{\lambda + \alpha }} u^{\sigma + \frac{1}{qn} - 1}\,du = k_{1}(\sigma). \end{aligned}$$

The lemma is proved. □

### Lemma 2

*If* $p > 1$, $\frac{1}{p} + \frac{1}{q} = 1$, $\sigma_{1} \in R$, $\beta > - 1$, $\mu = \lambda - \sigma > - \alpha $, *there exists a constant* $M_{2}$, *such that*, *for any non*-*negative measurable functions* $f(x)$ *and* $g(y)$ *in* (0, ∞), *the following inequality*:

17 $$\begin{aligned}& \int_{0}^{\infty } g(y) \biggl[ \int_{\frac{1}{y}}^{\infty } \frac{ ( \min \{ xy,1 \} ) ^{\alpha } \vert \ln xy \vert ^{\beta }}{ ( \max \{ xy,1 \} ) ^{\lambda + \alpha }} f(x)\,dx \biggr]\,dy \\& \quad \le M_{2} \biggl[ \int_{0}^{\infty } x^{p ( 1 - \sigma ) - 1}f ^{p}(x) \,dx \biggr] ^{\frac{1}{p}} \biggl[ \int_{0}^{\infty } y^{q ( 1 - \sigma_{1} ) - 1}g^{q}(y)\,dy \biggr] ^{\frac{1}{q}} \end{aligned}$$

*holds true*, *then we have* $\sigma_{1} = \sigma $, *and then* $M_{2} \ge k_{1}(\mu)$.

### Proof

If $\sigma_{1} < \sigma $, then, for $n \ge \frac{1}{ \sigma - \sigma_{1}}$ ($n \in \mathbf{N}$), we set two functions $\tilde{f}_{n}(x)$ and $\tilde{g}_{n}(y)$ as in Lemma 1, and find

$$ \tilde{J}_{1} = \biggl[ \int_{0}^{\infty } x^{p ( 1 - \sigma ) - 1}\tilde{f}_{n}^{p}(x) \,dx \biggr] ^{\frac{1}{p}} \biggl[ \int_{0}^{ \infty } y^{q ( 1 - \sigma_{1} ) - 1}\tilde{g}_{n}^{q}(y) \,dy \biggr] ^{\frac{1}{q}} = n. $$

Setting $u = xy$, we obtain

$$\begin{aligned} \tilde{I}_{2}&: = \int_{0}^{\infty } \tilde{g}_{n}(y) \biggl( \int_{\frac{1}{y}}^{\infty } \frac{ ( \min \{ xy,1 \} ) ^{\alpha } \vert \ln xy \vert ^{\beta }}{ ( \max \{ xy,1 \} ) ^{\lambda + \alpha }} \tilde{f}_{n}(x)\,dx \biggr)\,dy \\ &= \int_{0}^{1} \biggl( \int_{\frac{1}{y}}^{\infty } \frac{ (\min \{ xy,1 \} ) ^{\alpha } ( \ln xy ) ^{\beta }}{ ( \max \{ xy,1 \} ) ^{\lambda + \alpha }} x^{\sigma - \frac{1}{pn} - 1}\,dx \biggr) y^{\sigma_{1} + \frac{1}{qn} - 1}\,dy \\ &= \int_{0}^{1} y^{ ( \sigma_{1} - \sigma ) + \frac{1}{n} - 1}\,dy \int_{1}^{\infty } \frac{ ( \min \{ u,1 \} ) ^{ \alpha } ( \ln u ) ^{\beta }}{ ( \max \{ u,1 \} ) ^{\lambda + \alpha }} u^{\sigma - \frac{1}{pn} - 1} \,du, \end{aligned}$$

and then by (17), we obtain

18 $$\begin{aligned}& \int_{0}^{1} y^{ ( \sigma_{1} - \sigma ) + \frac{1}{n} - 1}\,dy \int_{1}^{\infty } \frac{ ( \min \{ u,1 \} ) ^{ \alpha } ( \ln u ) ^{\beta }}{ ( \max \{ u,1 \} ) ^{\lambda + \alpha }} u^{\sigma - \frac{1}{pn} - 1}\,du \\& \quad = \tilde{I}_{2} \le M_{2}\tilde{J}_{1} = M_{2}n < \infty . \end{aligned}$$

Since $( \sigma_{1} - \sigma ) + \frac{1}{n} \le 0$, it follows that $\int_{1}^{\infty } y^{ ( \sigma_{1} - \sigma ) + \frac{1}{n} - 1}\,dy = \infty $. By (18), in view of

$$ \int_{1}^{ \infty } \frac{ ( \min \{ u,1 \} ) ^{\alpha } ( \ln u ) ^{\beta }}{ ( \max \{ u,1 \} ) ^{\lambda + \alpha }} u^{\sigma - \frac{1}{pn} - 1} \,du > 0, $$

we have $\infty < \infty$, which is a contradiction.

If $\sigma_{1} > \sigma $, then, for $n \ge \frac{1}{\sigma_{1} -\sigma}$ ($n \in \mathbf{N}$), we set two functions $f_{n}(x)$ and $g_{n}(y)$ as in Lemma 1, and we find

$$ J_{1} = \biggl[ \int_{0}^{\infty } x^{p ( 1 - \sigma ) - 1}f _{n}^{p}(x) \,dx \biggr] ^{\frac{1}{p}} \biggl[ \int_{0}^{\infty } y^{q ( 1 - \sigma_{1} ) - 1}g_{n}^{q}(y) \,dy \biggr] ^{\frac{1}{q}} = n. $$

Setting $u = xy$, we obtain

$$\begin{aligned} I_{2} &: = \int_{0}^{\infty } f_{n}(x) \biggl( \int_{\frac{1}{x}}^{\infty } \frac{ ( \min \{ xy,1 \} ) ^{\alpha } \vert \ln xy \vert ^{\beta }}{ ( \max \{ xy,1 \} ) ^{\lambda + \alpha }} g_{n}(y)\,dy \biggr)\,dx \\ &= \int_{0}^{1} \biggl[ \int_{\frac{1}{x}}^{\infty } \frac{ (\min \{ xy,1 \} ) ^{\alpha } ( \ln xy ) ^{\beta }}{ ( \max \{ xy,1 \} ) ^{\lambda + \alpha }} y^{\sigma_{1} - \frac{1}{qn} - 1}\,dy \biggr] x^{\sigma + \frac{1}{pn} - 1}\,dx \\ &= \int_{0}^{1} x^{ ( \sigma - \sigma_{1} ) + \frac{1}{n} - 1}\,dx \int_{1}^{\infty } \frac{ ( \min \{ u,1 \} ) ^{ \alpha } ( \ln u ) ^{\beta }}{ ( \max \{ u,1 \} ) ^{\lambda + \alpha }} u^{\sigma_{1} - \frac{1}{qn} - 1} \,du, \end{aligned}$$

and then by Fubini theorem (*cf.* [20]) and (17), we have

19 $$\begin{aligned}& \int_{0}^{1} x^{ ( \sigma - \sigma_{1} ) + \frac{1}{n} - 1}\,dx \int_{1}^{\infty } \frac{ ( \min \{ u,1 \} ) ^{ \alpha } ( \ln u ) ^{\beta }}{ ( \max \{ u,1 \} ) ^{\lambda + \alpha }} u^{\sigma_{1} - \frac{1}{qn} - 1}\,du \\& \quad = I_{2} = \int_{0}^{\infty } g_{n}(y) \biggl( \int_{\frac{1}{y}}^{ \infty } \frac{ ( \min \{ xy,1 \} ) ^{\alpha } ( \ln xy ) ^{\beta }}{ ( \max \{ xy,1 \} ) ^{\lambda + \alpha }} f_{n}(x)\,dx \biggr)\,dy \\& \quad\le M_{2}J_{1} = M_{2}n. \end{aligned}$$

Since $( \sigma - \sigma_{1} ) + \frac{1}{n} \le 0$, it follows that $\int_{0}^{1} x^{ ( \sigma - \sigma_{1} ) + \frac{1}{n} - 1}\,dx = \infty $. By (19), in view of

$$ \int_{1}^{\infty } \frac{ ( \min \{ u,1 \} ) ^{ \alpha } ( \ln u ) ^{\beta }}{ ( \max \{ u,1 \} ) ^{\lambda + \alpha }} u^{\sigma_{1} - \frac{1}{qn} - 1}\,du > 0, $$

we have $\infty < \infty $, which is a contradiction.

Hence, we conclude that $\sigma_{1} = \sigma $.

For $\sigma_{1} = \sigma $, we reduce (19) as follows:

20 $$ M_{2} \ge \int_{1}^{\infty } \frac{ ( \min \{ u,1 \} ) ^{\alpha } ( \ln u ) ^{\beta }}{ ( \max \{ u,1 \} ) ^{\lambda + \alpha }} u^{\sigma - \frac{1}{qn} - 1} \,du. $$

Since

$$ \biggl\{ \frac{ ( \min \{ u,1 \} ) ^{\alpha } ( \ln u ) ^{\beta }}{ ( \max \{ u,1 \} ) ^{\lambda + \alpha }} u^{\sigma - \frac{1}{qn} - 1} \biggr\} _{n = 1} ^{\infty } $$

is non-negative and increasing in $[ 1, \infty)$, still by the Levi theorem (*cf.* [20]), we have

$$\begin{aligned} M_{2} &\ge \lim_{n \to \infty } \int_{1}^{\infty } \frac{ ( \min \{ u,1 \} ) ^{\alpha } ( \ln u ) ^{\beta }}{ ( \max \{ u,1 \} ) ^{\lambda + \alpha }} u^{ \sigma - \frac{1}{qn} - 1}\,du \\ & = \int_{1}^{\infty } \lim_{n \to \infty } \frac{ ( \min \{ u,1 \} ) ^{\alpha } ( \ln u ) ^{\beta }}{ (\max \{ u,1 \} ) ^{\lambda + \alpha }} u^{\sigma - \frac{1}{qn} - 1}\,du = k_{1}(\mu). \end{aligned}$$

The lemma is proved. □

## Main results and corollaries

### Theorem 1

*If* $p > 1$, $\frac{1}{p} + \frac{1}{q} = 1$, $\sigma _{1} \in R$, $\beta > - 1$, $\sigma > - \alpha $, *then the following conditions are equivalent*:

(i) *There exists a constant* $M_{1}$, *such that*, *for any* $f(x) \ge 0$, *satisfying*
  $$ 0 < \int_{0}^{\infty } x^{p ( 1 - \sigma ) - 1}f^{p}(x)\,dx < \infty , $$
  *we have the following Hardy*-*type integral inequality of the first kind with the non*-*homogeneous kernel*:
  21 $$\begin{aligned} J&: = \biggl\{ \int_{0}^{\infty } y^{p\sigma_{1} - 1} \biggl[ \int_{0} ^{\frac{1}{y}} \frac{ ( \min \{ xy,1 \} ) ^{ \alpha } \vert \ln xy \vert ^{\beta }}{ ( \max \{ xy,1 \} ) ^{\lambda + \alpha }} f(x)\,dx \biggr] ^{p}\,dy \biggr\} ^{\frac{1}{p}} \\ &< M_{1} \biggl[ \int_{0}^{\infty } x^{p ( 1 - \sigma ) - 1}f ^{p}(x) \,dx \biggr] ^{\frac{1}{p}}. \end{aligned}$$
(ii) *There exists a constant* $M_{1}$, *such that*, *for any* $f(x)$, $g(y) \ge 0$, *satisfying* $0 < \int_{0}^{\infty } x^{p ( 1 - \sigma ) - 1}f^{p}(x)\,dx < \infty $, *and* $0 < \int_{0}^{\infty } y^{q ( 1 - \sigma_{1} ) - 1}g^{q}(y)\,dy < \infty $, *we have the following inequality*:
  22 $$\begin{aligned} I&: = \int_{0}^{\infty } g(y) \biggl[ \int_{0}^{\frac{1}{y}} \frac{ (\min \{ xy,1 \} ) ^{\alpha } \vert \ln xy \vert ^{\beta }}{ ( \max \{ xy,1 \} ) ^{\lambda + \alpha }} f(x)\,dx \biggr] \,dy \\ &< M_{1} \biggl[ \int_{0}^{\infty } x^{p ( 1 - \sigma ) - 1}f ^{p}(x) \,dx \biggr] ^{\frac{1}{p}} \biggl[ \int_{0}^{\infty } y^{q ( 1 - \sigma_{1} ) - 1}g^{q}(y)\,dy \biggr] ^{\frac{1}{q}}. \end{aligned}$$
(iii) $\sigma_{1} = \sigma $.

*If condition* (iii) *holds true*, *then* $M_{1} \ge k_{1}(\sigma)$ *and the constant factor*

$$ M_{1} = k_{1}(\sigma) = \frac{\Gamma (\beta + 1)}{ ( \sigma + \alpha ) ^{\beta + 1}} $$

*in* (21) *and* (22) *is the best possible*.

### Proof

(i)⇒(ii). By Hölder’s inequality (*cf.* [21]), we have

23 $$\begin{aligned} I &= \int_{0}^{\infty } \biggl[ y^{\sigma_{1} - \frac{1}{p}} \int_{0} ^{\frac{1}{y}} \frac{ ( \min \{ xy,1 \} ) ^{ \alpha } \vert \ln xy \vert ^{\beta }}{ ( \max \{ xy,1 \} ) ^{\lambda + \alpha }} f(x)\,dx \biggr] \bigl( y^{ \frac{1}{p} - \sigma_{1}}g(y) \bigr)\,dy \\ &\le J \biggl[ \int_{0}^{\infty } y^{q ( 1 - \sigma_{1} ) - 1}g ^{q}(y) \,dy \biggr] ^{\frac{1}{q}}. \end{aligned}$$

Then by (21), we have (22).

(ii)⇒(iii). By Lemma 1, we have $\sigma_{1} = \sigma $.

(iii)⇒(i). Setting $u = xy$, we obtain the following weight function:

24 $$\begin{aligned} \omega_{1}(\sigma ,y)&: = y^{\sigma } \int_{0}^{\frac{1}{y}} \frac{ ( \min \{ xy,1 \} ) ^{\alpha } \vert \ln xy \vert ^{\beta }}{ ( \max \{ xy,1 \} ) ^{\lambda + \alpha }} x^{\sigma - 1} \,dx \\ &= \int_{0}^{1} \frac{ ( \min \{ u,1 \} ) ^{\alpha } ( - \ln u ) ^{\beta }}{ ( \max \{ u,1 \} ) ^{\lambda + \alpha }} u^{\sigma - 1} \,du = k_{1}(\sigma)\quad (y > 0). \end{aligned}$$

By Hölder’s inequality with weight and (24), for $y \in (0,\infty)$, we have

25 $$\begin{aligned}& \biggl[ \int_{0}^{\frac{1}{y}} \frac{ ( \min \{ xy,1 \} ) ^{\alpha } \vert \ln xy \vert ^{\beta }}{ ( \max \{ xy,1 \} ) ^{\lambda + \alpha }} f(x)\,dx \biggr] ^{p} \\& \quad = \biggl\{ \int_{0}^{\frac{1}{y}} \frac{ ( \min \{ xy,1 \} ) ^{\alpha } \vert \ln xy \vert ^{\beta }}{ ( \max \{ xy,1 \} ) ^{\lambda + \alpha }} \biggl[ \frac{y^{ (\sigma - 1 ) / p}}{x^{ ( \sigma - 1 ) / q}}f(x) \biggr] \biggl[ \frac{x^{ ( \sigma - 1 ) / q}}{y^{ ( \sigma - 1 ) / p}} \biggr]\,dx \biggr\} ^{p} \\& \quad \le \int_{0}^{\frac{1}{y}} \frac{ ( \min \{ xy,1 \} ) ^{\alpha } \vert \ln xy \vert ^{\beta }}{ ( \max \{ xy,1 \} ) ^{\lambda + \alpha }} \frac{y^{\sigma - 1}f^{p}(x)}{x ^{ ( \sigma - 1 ) p / q}} \,dx \\& \quad \quad {}\times \biggl[ \int_{0}^{\frac{1}{y}} \frac{ ( \min \{ xy,1 \} ) ^{\alpha } \vert \ln xy \vert ^{\beta }}{ (\max \{ xy,1 \} ) ^{\lambda + \alpha }} \frac{x^{ \sigma - 1}}{y^{ ( \sigma - 1 ) q / p}} \,dx \biggr] ^{p - 1} \\& \quad = \biggl[ \frac{\omega_{1}(\sigma ,y)}{y^{q ( \sigma - 1 ) + 1}} \biggr] ^{p - 1} \int_{0}^{\frac{1}{y}} \frac{ ( \min \{ xy,1 \} ) ^{\alpha } \vert \ln xy \vert ^{\beta }}{ (\max \{ xy,1 \} ) ^{\lambda + \alpha }} \frac{y^{ \sigma - 1}}{x^{ ( \sigma - 1 ) p / q}}f^{p}(x)\,dx \\& \quad = \bigl( k_{1}(\sigma) \bigr) ^{p - 1}y^{ - p\sigma + 1} \int_{0}^{ \frac{1}{y}} \frac{ ( \min \{ xy,1 \} ) ^{\alpha } \vert \ln xy \vert ^{\beta }}{ ( \max \{ xy,1 \} ) ^{\lambda + \alpha }} \frac{y^{\sigma - 1}}{x^{ ( \sigma - 1 ) p / q}}f^{p}(x)\,dx. \end{aligned}$$

If (25) obtains the form of equality for a $y \in (0,\infty)$, then (*cf.* [21]), there exist constants *A* and *B*, such that they are not all zero, and

$$ A\frac{y^{\sigma - 1}}{x^{ ( \sigma - 1 ) p / q}}f^{p}(x) = B\frac{x ^{\sigma - 1}}{y^{ ( \sigma - 1 ) q / p}}\quad\mbox{a.e. in } \mathbf{R}_{ +}. $$

We suppose that $A \ne 0$ (otherwise $B = A = 0$). It follows that

$$ x^{p ( 1 - \sigma ) - 1}f^{p}(x) = y^{q ( \sigma - 1 ) } \frac{B}{Ax}\quad \mbox{a.e. in } \mathbf{R}_{+}, $$

which contradicts the fact that $0 < \int_{0}^{\infty } x^{p ( 1 - \sigma ) - 1}f^{p}(x)\,dx < \infty $. Hence, (25) assumes the form of strict inequality. Hence, for $\sigma_{1} = \sigma $, by (25) and by the Fubini theorem (*cf.* [20]), we obtain

$$\begin{aligned} J &< \bigl( k_{1}(\sigma) \bigr) ^{\frac{1}{q}} \biggl\{ \int_{0}^{ \infty } \biggl[ \int_{0}^{\frac{1}{y}} \frac{ ( \min \{ xy,1 \} ) ^{\alpha } \vert \ln xy \vert ^{\beta }}{ (\max \{ xy,1 \} ) ^{\lambda + \alpha }} \frac{y^{ \sigma - 1}f^{p}(x)}{x^{ ( \sigma - 1 ) p / q}} \,dx \biggr]\,dy \biggr\} ^{\frac{1}{p}} \\ & = \bigl( k_{1}(\sigma) \bigr) ^{\frac{1}{q}} \biggl\{ \int_{0}^{\infty } \biggl[ \int_{0}^{\frac{1}{x}} \frac{ ( \min \{ xy,1 \} ) ^{\alpha } \vert \ln xy \vert ^{\beta }}{ ( \max \{ xy,1 \} ) ^{\lambda + \alpha }} \frac{y^{\sigma - 1}\,dy}{x ^{ (\sigma - 1 ) ( p - 1 ) }} \biggr] f^{p}(x)\,dx \biggr\} ^{\frac{1}{p}} \\ &= \bigl( k_{1}(\sigma) \bigr) ^{\frac{1}{q}} \biggl[ \int_{0}^{\infty } \omega_{1}(\sigma ,x)x^{p ( 1 - \sigma ) - 1}f^{p}(x)\,dx \biggr] ^{\frac{1}{p}} \\ &= k_{1}(\sigma) \biggl[ \int_{0}^{\infty } x^{p ( 1 - \sigma ) - 1}f^{p}(x)\,dx \biggr] ^{\frac{1}{p}}. \end{aligned}$$

Setting $M_{1} \ge k_{1}(\sigma)$, (21) follows.

Therefore, conditions (i), (ii) and (iii) are equivalent.

When condition (iii) follows, if there exists a constant factor $M_{1} \ge k_{1}(\sigma)$, such that (22) is valid, then by Lemma 1, we have $M_{1} \ge k_{1}(\sigma)$. Hence, the constant factor $M_{1} = k_{1}(\sigma)$ in (22) is the best possible. The constant factor $M_{1} = k_{1}(\sigma)$ in (21) is still the best possible. Otherwise, by (23) (for $\sigma_{1} = \sigma $), we can conclude that the constant factor $M_{1} = k_{1}(\sigma)$ in (22) is not the best possible. □

Setting $y = \frac{1}{Y}$, $G(Y) = Y^{\lambda - 2}g(\frac{1}{Y})$, $\mu_{1} = \lambda - \sigma_{1}$ in Theorem 1, then replacing *Y* by *y* and $G(Y)$ by $g(y)$, we have

### Corollary 1

*If* $p > 1$, $\frac{1}{p} + \frac{1}{q} = 1$, $\mu_{1} \in R$, $\beta > - 1$, $\sigma = \lambda - \mu > - \alpha $, *then the following conditions are equivalent*:

(i) *There exists a constant* $M_{1}$, *such that*, *for any* $f(x) \ge 0$, *satisfying*
  $$ 0 < \int_{0}^{\infty } x^{p ( 1 - \sigma ) - 1}f^{p}(x)\,dx < \infty , $$
  *we have the following Hardy*-*type inequality of the first kind with the homogeneous kernel*:
  26 $$\begin{aligned}& \biggl\{ \int_{0}^{\infty } y^{p\mu_{1} - 1} \biggl[ \int_{0}^{y} \frac{ (\min \{ x,y \} ) ^{\alpha } \vert \ln ( x / y ) \vert ^{\beta }}{ ( \max \{ x,y \} ) ^{\lambda + \alpha }} f(x)\,dx \biggr] ^{p}\,dy \biggr\} ^{\frac{1}{p}} \\& \quad < M_{1} \biggl[ \int_{0}^{\infty } x^{p ( 1 - \sigma ) - 1}f ^{p}(x) \,dx \biggr] ^{\frac{1}{p}}. \end{aligned}$$
(ii) *There exists a constant* $M_{1}$, *such that*, *for any* $f(x)$, $g(y) \ge 0$, *satisfying* $0 < \int_{0}^{\infty } x^{p ( 1 - \sigma ) - 1}f^{p}(x)\,dx < \infty $, *and* $0 < \int_{0}^{\infty } y^{q ( 1 - \mu_{1} ) - 1}g^{q}(y)\,dy < \infty $, *we have the following inequality*:
  27 $$\begin{aligned}& \int_{0}^{\infty } g(y) \biggl[ \int_{0}^{y} \frac{ ( \min \{ x,y \} ) ^{\alpha } \vert \ln ( x / y ) \vert ^{\beta }}{ ( \max \{ x,y \} ) ^{\lambda + \alpha }} f(x)\,dx \biggr] \,dy \\& \quad< M_{1} \biggl[ \int_{0}^{\infty } x^{p ( 1 - \sigma ) - 1}f ^{p}(x) \,dx \biggr] ^{\frac{1}{p}} \biggl[ \int_{0}^{\infty } y^{q ( 1 - \mu_{1} ) - 1}g^{q}(y) \,dy \biggr] ^{\frac{1}{q}}. \end{aligned}$$
(iii) $\mu_{1} = \mu $.

*If condition* (iii) *holds true*, *then we have* $M_{1} \ge k_{1}(\sigma)$, *and the constant* $M_{1} = k_{1}(\sigma)$ *in* (26) *and* (27) *is the best possible*.

### Remark 2

On the other hand, setting $y = \frac{1}{Y}$, $G(Y) = Y^{\lambda - 2}g(\frac{1}{Y})$, $\sigma_{1} = \lambda - \mu_{1}$ in Corollary 1, then replacing *Y* by *y* and $G(Y)$ by $g(y)$, we have Theorem 1. Hence, Theorem 1 and Corollary 1 are equivalent.

Similarly, we obtain the following weight function:

$$\begin{aligned} \omega_{2}(\sigma ,y)&: = y^{\sigma } \int_{\frac{1}{y}}^{\infty } \frac{ (\min \{ xy,1 \} ) ^{\alpha } \vert \ln xy \vert ^{\beta }}{ ( \max \{ xy,1 \} ) ^{\lambda + \alpha }} x^{\sigma - 1}\,dx \\ &= \int_{1}^{\infty } \frac{ ( \min \{ u,1 \} ) ^{\alpha } \vert \ln u \vert ^{\beta }}{ ( \max \{ u,1 \} ) ^{\lambda + \alpha }} u^{\sigma - 1}\,du = k_{1}(\mu) \quad (y > 0), \end{aligned}$$

and then in view of Lemma 2 and in the same way, we have

### Theorem 2

*If* $p > 1$, $\frac{1}{p} + \frac{1}{q} = 1$, $\sigma _{1} \in R$, $\beta > - 1$, $\mu = \lambda - \sigma > - \alpha $, *then the following conditions are equivalent*:

(i) *There exists a constant* $M_{2}$, *such that*, *for any* $f(x) \ge 0$, *satisfying*
  $$ 0 < \int_{0}^{\infty } x^{p ( 1 - \sigma ) - 1}f^{p}(x)\,dx < \infty , $$
  *we have the following Hardy*-*type inequality of the second kind with the non*-*homogeneous kernel*:
  28 $$\begin{aligned}& \biggl\{ \int_{0}^{\infty } y^{p\sigma_{1} - 1} \biggl[ \int_{ \frac{1}{y}}^{\infty } \frac{ ( \min \{ xy,1 \} ) ^{\alpha } \vert \ln xy \vert ^{\beta }}{ ( \max \{ xy,1 \} ) ^{\lambda + \alpha }} f(x)\,dx \biggr] ^{p}\,dy \biggr\} ^{\frac{1}{p}} \\& \quad < M_{2} \biggl[ \int_{0}^{\infty } x^{p ( 1 - \sigma ) - 1}f ^{p}(x) \,dx \biggr] ^{\frac{1}{p}}. \end{aligned}$$
(ii) *There exists a constant* $M_{2}$, *such that*, *for any* $f(x)$, $g(y) \ge 0$, *satisfying* $0 < \int_{0}^{\infty } x^{p ( 1 - \sigma ) - 1}f^{p}(x)\,dx < \infty $, *and* $0 < \int_{0}^{\infty } y^{q ( 1 - \sigma_{1} ) - 1}g^{q}(y)\,dy < \infty $, *we have the following inequality*:
  29 $$\begin{aligned}& \int_{0}^{\infty } g(y) \biggl[ \int_{\frac{1}{y}}^{\infty } \frac{ ( \min \{ xy,1 \} ) ^{\alpha } \vert \ln xy \vert ^{\beta }}{ ( \max \{ xy,1 \} ) ^{\lambda + \alpha }} f(x)\,dx \biggr]\,dy \\& \quad < M_{2} \biggl[ \int_{0}^{\infty } x^{p ( 1 - \sigma ) - 1}f ^{p}(x) \,dx \biggr] ^{\frac{1}{p}} \biggl[ \int_{0}^{\infty } y^{q ( 1 - \sigma_{1} ) - 1}g^{q}(y)\,dy \biggr] ^{\frac{1}{q}}. \end{aligned}$$
(iii) $\sigma_{1} = \sigma $.

*If condition* (iii) *holds true*, *then we have* $M_{2} \ge k_{1}(\mu)$ *and the constant factor*

$$ M_{2} = k_{1}(\mu) = \frac{\Gamma (\beta + 1)}{ ( \mu + \alpha ) ^{\beta + 1}} $$

*in* (28) *and* (29) *is the best possible*.

Setting $y = \frac{1}{Y}$, $G(Y) = Y^{\lambda - 2}g(\frac{1}{Y})$, $\mu_{1} = \lambda - \sigma_{1}$ in Theorem 2, then replacing *Y* by *y* and $G(Y)$ by $g(y)$, we have

### Corollary 2

*If* $p > 1$, $\frac{1}{p} + \frac{1}{q} = 1$, $\mu_{1} \in R$, $\beta > - 1$, $\mu = \lambda - \sigma > - \alpha $, *then the following conditions are equivalent*:

(i) *There exists a constant* $M_{2}$, *such that*, *for any* $f(x) \ge 0$, *satisfying*
  $$ 0 < \int_{0}^{\infty } x^{p ( 1 - \sigma ) - 1}f^{p}(x)\,dx < \infty , $$
  *we have the following Hardy*-*type inequality of the second kind with the homogeneous kernel*:
  30 $$\begin{aligned}& \biggl\{ \int_{0}^{\infty } y^{p\mu_{1} - 1} \biggl[ \int_{y}^{\infty } \frac{ ( \min \{ x,y \} ) ^{\alpha } \vert \ln ( x / y ) \vert ^{\beta }}{ ( \max \{ x,y \} ) ^{\lambda + \alpha }} f(x)\,dx \biggr] ^{p}\,dy \biggr\} ^{\frac{1}{p}} \\& \quad< M_{2} \biggl[ \int_{0}^{\infty } x^{p ( 1 - \sigma ) - 1}f ^{p}(x) \,dx \biggr] ^{\frac{1}{p}}. \end{aligned}$$
(ii) *There exists a constant* $M_{2}$, *such that*, *for any* $f(x)$, $g(y) \ge 0$, *satisfying* $0 < \int_{0}^{\infty } x^{p ( 1 - \sigma ) - 1}f^{p}(x)\,dx < \infty $, *and* $0 < \int_{0}^{\infty } y^{q ( 1 - \mu_{1} ) - 1}g^{q}(y)\,dy < \infty $, *we have the following inequality*:
  31 $$\begin{aligned}& \int_{0}^{\infty } g(y) \biggl[ \int_{y}^{\infty } \frac{ ( \min \{ x,y \} ) ^{\alpha } \vert \ln ( x / y ) \vert ^{\beta }}{ ( \max \{ x,y \} ) ^{\lambda + \alpha }} f(x)\,dx \biggr] \,dy \\& \quad< M_{2} \biggl[ \int_{0}^{\infty } x^{p ( 1 - \sigma ) - 1}f ^{p}(x) \,dx \biggr] ^{\frac{1}{p}} \biggl[ \int_{0}^{\infty } y^{q ( 1 - \mu_{1} ) - 1}g^{q}(y) \,dy \biggr] ^{\frac{1}{q}}. \end{aligned}$$
(iii) $\mu_{1} = \mu $.

*If condition* (iii) *holds true*, *then we have* $M_{2} \ge k_{1}(\mu)$, *and the constant* $M_{2} = k_{1}(\mu)$ *in* (30) *and* (31) *is the best possible*.

### Remark 3

Theorem 2 and Corollary 2 are still equivalent.

## Operator expressions

For $p > 1$, $\frac{1}{p} + \frac{1}{q} = 1$, *σ*, $\lambda > 0$, $\mu = \lambda - \sigma $, we set the following functions: $\varphi (x): = x ^{p ( 1 - \sigma ) - 1}$, $\psi (y): = y^{q ( 1 - \sigma ) - 1}$, $\phi (y): = y^{q ( 1 - \mu ) - 1}$, wherefrom, $\psi^{1 - p}(y) = y^{p \sigma - 1}$, $\phi^{1 - p}(y) = y^{p\mu - 1}$ ($x,y \in \mathbf{R}_{ +}$). Define the following real normed linear spaces:

$$ L_{p,\varphi } (\mathbf{R}_{ +}): = \biggl\{ f:\Vert f \Vert _{p,\varphi }: = \biggl( \int_{0}^{\infty } \varphi(x) \bigl\vert f(x) \bigr\vert ^{p}\,dx \biggr) ^{\frac{1}{p}} < \infty \biggr\} , $$

wherefrom

$$\begin{aligned}& L_{q,\psi } (\mathbf{R}_{ +}): = \biggl\{ g:\Vert g \Vert _{q,\psi }: = \biggl( \int_{0}^{\infty } \psi (y) \bigl\vert g(y) \bigr\vert ^{q}\,dy \biggr) ^{\frac{1}{q}} < \infty \biggr\} , \\& L_{q,\phi } (\mathbf{R}_{ +}): = \biggl\{ g:\Vert g \Vert _{q,\phi }: = \biggl( \int_{0}^{\infty } \phi (y) \bigl\vert g(y) \bigr\vert ^{q}\,dy \biggr) ^{\frac{1}{q}} < \infty \biggr\} , \\& L_{p,\psi^{1 - p}}(\mathbf{R}_{ +}) = \biggl\{ h:\Vert h \Vert _{p,\psi^{1 - p}} = \biggl( \int_{0}^{\infty } \psi^{1 - p}(y) \bigl\vert h(y) \bigr\vert ^{p}\,dy \biggr) ^{\frac{1}{p}} < \infty \biggr\} , \\& L_{q,\phi^{1 - p}}(\mathbf{R}_{ +}) = \biggl\{ h:\Vert h \Vert _{p,\phi^{1 - p}} = \biggl( \int_{0}^{\infty } \phi^{1 - p}(y) \bigl\vert h(y) \bigr\vert ^{p}\,dy \biggr) ^{\frac{1}{p}} < \infty \biggr\} . \end{aligned}$$

(a) In view of Theorem 1 ($\sigma_{1} = \sigma$), for $f \in L_{p, \varphi} (\mathbf{R}_{ +})$, setting

$$ h_{1}(y): = \int_{0}^{\frac{1}{y}} \frac{ ( \min \{ xy,1 \} ) ^{\alpha } \vert \ln xy \vert ^{\beta }}{ (\max \{ xy,1 \} ) ^{\lambda + \alpha }} f(x)\,dx \quad (y \in \mathbf{R}_{ +}), $$

by (21), we have

32 $$ \Vert h_{1} \Vert _{p,\psi^{1 - p}} = \biggl[ \int_{0}^{\infty } \psi^{1 - p}(y)h_{1}^{p}(y) \,dy \biggr] ^{\frac{1}{p}} < M_{1}\Vert f \Vert _{p,\varphi} < \infty . $$

### Definition 1

Define a Hardy-type integral operator of the first kind with the non-homogeneous kernel $T_{1}^{(1)}:L_{p,\varphi} (\mathbf{R}_{ +}) \to L_{p,\psi^{1 - p}}(\mathbf{R}_{ +})$ as follows: For any $f \in L_{p,\varphi} (\mathbf{R}_{ +})$, there exists a unique representation $T_{1}^{(1)}f = h_{1} \in L_{p,\psi ^{1 - p}}(\mathbf{R}_{ +})$, satisfying for any $y \in \mathbf{R}_{ +}$, $T_{1}^{(1)}f(y) = h_{1}(y)$.

In view of (32), it follows that $\Vert T_{1}^{(1)}f \Vert _{p, \psi^{1 - p}} = \Vert h_{1} \Vert _{p,\psi^{1 - p}} \le M_{1} \Vert f \Vert _{p,\varphi}$, and then the operator $T_{1}^{(1)}$ is bounded satisfying

$$ \bigl\Vert T_{1}^{(1)} \bigr\Vert = \sup _{f(\ne \theta) \in L_{p,\varphi} (R_{ +} )}\frac{\Vert T_{1} ^{(1)}f \Vert _{p,\psi^{1 - p}}}{\Vert f \Vert _{p,\varphi}} \le M_{1}. $$

If we define the formal inner product of $T_{1}^{(1)}f$ and *g* as follows:

$$ \bigl( T_{1}^{(1)}f,g \bigr) : = \int_{0}^{\infty } \biggl( \int_{0} ^{\frac{1}{y}} \frac{ ( \min \{ xy,1 \} ) ^{ \alpha } \vert \ln xy \vert ^{\beta }}{ ( \max \{ xy,1 \} ) ^{\lambda + \alpha }} f(x)\,dx \biggr) g(y)\,dy, $$

then we can rewrite Theorem 1 (for $\sigma_{1} = \sigma $) as follows.

### Theorem 3

*If* $p > 1$, $\frac{1}{p} + \frac{1}{q} = 1$, $\beta > - 1$, $\sigma > - \alpha $, *then the following conditions are equivalent*:

(i) *There exists a constant* $M_{1}$, *such that*, *for any* $f(x) \ge 0,f \in L_{p,\varphi} (\mathbf{R}_{ +}),\Vert f \Vert _{p,\varphi} > 0$, *we have the following inequality*:
  33 $$ \bigl\Vert T_{1}^{(1)}f \bigr\Vert _{p,\psi^{1 - p}} < M_{1}\Vert f \Vert _{p,\varphi}. $$
(ii) *There exists a constant* $M_{1}$, *such that*, *for any* $f(x)$, $g(y)\ge 0$, $f \in L_{p,\varphi} (\mathbf{R}_{ +})$, $g \in L_{q,\psi } (\mathbf{R}_{ +}),\Vert f \Vert _{p,\varphi}, \Vert g \Vert _{q,\psi } > 0$, *we have the following inequality*:
  34 $$ \bigl( T_{1}^{(1)}f,g \bigr) < M_{1}\Vert f \Vert _{p,\varphi} \Vert g \Vert _{q,\psi }. $$

*We still have* $\Vert T_{1}^{(1)} \Vert = k_{1}(\sigma) \le M _{1}$.

(b) In view of Corollary 1 ($\mu_{1} = \mu$), for $f \in L_{p,\varphi} (\mathbf{R}_{ +})$, setting

$$ h_{2}(y): = \int_{0}^{y} \frac{ ( \min \{ x,y \} ) ^{\alpha } \vert \ln ( x / y ) \vert ^{\beta }}{ (\max \{ x,y \} ) ^{\lambda + \alpha }} f(x)\,dx \quad (y \in \mathbf{R}_{ +}), $$

by (26), we have

35 $$ \Vert h_{2} \Vert _{p,\phi^{1 - p}} = \biggl[ \int_{0}^{\infty } \phi^{1 - p}(y)h_{2}^{p}(y) \,dy \biggr] ^{\frac{1}{p}} < M_{1}\Vert f \Vert _{p,\varphi} < \infty . $$

### Definition 2

Define a Hardy-type integral operator of the first kind with the homogeneous kernel $T_{1}^{(2)}:L_{p,\varphi} (\mathbf{R}_{ +}) \to L_{p,\phi^{1 - p}}(\mathbf{R}_{ +})$ as follows: For any $f \in L_{p,\varphi} (\mathbf{R}_{ +})$, there exists a unique representation $T_{1}^{(2)}f = h_{2} \in L_{p, \phi^{1 - p}}(\mathbf{R}_{ +})$, satisfying for any $y \in \mathbf{R}_{ +}$, $T_{1}^{(2)}f(y) = h_{2}(y)$.

In view of (35), it follows that $\Vert T_{1}^{(2)}f \Vert _{p, \phi^{1 - p}} = \Vert h_{2} \Vert _{p,\phi^{1 - p}} \le M _{1}\Vert f \Vert _{p,\varphi}$, and then the operator $T_{1}^{(2)}$ is bounded satisfying

$$ \bigl\Vert T_{1}^{(2)} \bigr\Vert = \sup _{f(\ne \theta) \in L_{p,\varphi} (R_{ +} )}\frac{\Vert T_{1} ^{(2)}f \Vert _{p,\phi^{1 - p}}}{\Vert f \Vert _{p,\varphi}} \le M_{1}. $$

If we define the formal inner product of $T_{1}^{(2)}f$ and *g* as follows:

$$ \bigl( T_{1}^{(2)}f,g \bigr) : = \int_{0}^{\infty } \biggl( \int_{0} ^{y} \frac{ ( \min \{ x,y \} ) ^{\alpha } \vert \ln ( x / y ) \vert ^{\beta }}{ ( \max \{ x,y \} ) ^{\lambda + \alpha }} f(x)\,dx \biggr) g(y)\,dy, $$

then we can rewrite Corollary 1 (for $\mu_{1} = \mu $) as follows.

### Corollary 3

*If* $p > 1,\frac{1}{p} + \frac{1}{q} = 1,\beta > - 1,\sigma = \lambda - \mu > - \alpha $, *then the following conditions are equivalent*:

(i) *There exists a constant* $M_{1}$, *such that*, *for any* $f(x) \ge 0,f \in L_{p,\varphi} (\mathbf{R}_{ +}),\Vert f \Vert _{p,\varphi} > 0$, *we have the following inequality*:
  36 $$ \bigl\Vert T_{1}^{(2)}f \bigr\Vert _{p,\phi^{1 - p}} < M_{1}\Vert f \Vert _{p,\varphi}. $$
(ii) *There exists a constant* $M_{1}$, *such that*, *for any* $f(x),g(y) \ge 0,f \in L_{p,\varphi} (\mathbf{R}_{ +}),g \in $
$L_{q,\phi } (\mathbf{R}_{ +}),\Vert f \Vert _{p,\varphi}, \Vert g \Vert _{q,\phi } > 0$, *we have the following inequality*:
  37 $$ \bigl( T_{1}^{(2)}f,g \bigr) < M_{1}\Vert f \Vert _{p,\varphi} \Vert g \Vert _{q,\phi }. $$

*We still have* $\Vert T_{1}^{(2)} \Vert = k_{1}(\sigma) \le M _{1}$.

### Remark 4

Theorem 3 and Corollary 3 are equivalent.

(c) In view of Theorem 2 ($\sigma_{1} = \sigma$), for $f \in L_{p, \varphi} (\mathbf{R}_{ +})$, setting

$$ H_{1}(y): = \int_{\frac{1}{y}}^{\infty } \frac{ ( \min \{ xy,1 \} ) ^{\alpha } \vert \ln xy \vert ^{\beta }}{ (\max \{ xy,1 \} ) ^{\lambda + \alpha }} f(x)\,dx \quad (y \in \mathbf{R}_{ +}), $$

by (28), we have

38 $$ \Vert H_{1} \Vert _{p,\psi^{1 - p}} = \biggl[ \int_{0}^{\infty } \psi^{1 - p}(y)H_{1}^{p}(y) \,dy \biggr] ^{\frac{1}{p}} < M_{2}\Vert f \Vert _{p,\varphi} < \infty . $$

### Definition 3

Define a Hardy-type integral operator of the second kind with the non-homogeneous kernel $T_{2}^{(1)}:L_{p,\varphi} (\mathbf{R}_{ +}) \to L_{p,\psi^{1 - p}}(\mathbf{R}_{ +})$ as follows: For any $f \in L_{p,\varphi} (\mathbf{R}_{ +})$, there exists a unique representation $T_{2}^{(1)}f = H_{1} \in L_{p,\psi ^{1 - p}}(\mathbf{R}_{ +})$, satisfying for any $y \in \mathbf{R}_{ +}$, $T_{2}^{(1)}f(y) = H_{1}(y)$.

In view of (38), it follows that $\Vert T_{2}^{(1)}f \Vert _{p, \psi^{1 - p}} = \Vert H_{1} \Vert _{p,\psi^{1 - p}} \le M_{2} \Vert f \Vert _{p,\varphi}$, and then the operator $T_{2}^{(1)}$ is bounded satisfying

$$ \bigl\Vert T_{2}^{(1)} \bigr\Vert = \sup _{f(\ne \theta) \in L_{p,\varphi} (R_{ +} )}\frac{\Vert T_{2} ^{(1)}f \Vert _{p,\psi^{1 - p}}}{\Vert f \Vert _{p,\varphi}} \le M_{2}. $$

If we define the formal inner product of $T_{2}^{(1)}f$ and *g* as follows:

$$ \bigl( T_{2}^{(1)}f,g \bigr) : = \int_{0}^{\infty } \biggl( \int_{\frac{1}{y}}^{\infty } \frac{ ( \min \{ xy,1 \} ) ^{\alpha } \vert \ln xy \vert ^{\beta }}{ ( \max \{ xy,1 \} ) ^{\lambda + \alpha }} f(x)\,dx \biggr) g(y)\,dy, $$

then we can rewrite Theorem 2 (for $\sigma_{1} = \sigma $) as follows.

### Theorem 4

*If* $p > 1$, $\frac{1}{p} + \frac{1}{q} = 1$, $\beta > - 1$, $\mu = \lambda - \sigma > - \alpha $, *then the following conditions are equivalent*:

(i) *There exists a constant* $M_{2}$, *such that*, *for any* $f(x) \ge 0$, $f \in L_{p,\varphi} (\mathbf{R}_{ +})$, $\Vert f \Vert _{p,\varphi} > 0$, *we have the following inequality*:
  39 $$ \bigl\Vert T_{2}^{(1)}f \bigr\Vert _{p,\psi^{1 - p}} < M_{2}\Vert f \Vert _{p,\varphi}. $$
(ii) *There exists a constant* $M_{2}$, *such that*, *for any* $f(x)$, $g(y) \ge 0$, $f \in L_{p,\varphi} (\mathbf{R}_{ +})$, $g \in L_{q,\psi } (\mathbf{R}_{ +})$, $\Vert f \Vert _{p,\varphi}, \Vert g \Vert _{q,\psi } > 0$, *we have the following inequality*:
  40 $$ \bigl( T_{2}^{(1)}f,g \bigr) < M_{2} \Vert f \Vert _{p,\varphi} \Vert g \Vert _{q,\psi }. $$

*We still have* $\Vert T_{2}^{(1)} \Vert = k_{1}(\mu) \le M_{2}$.

(d) In view of Corollary 2 ($\mu_{1} = \mu$), for $f \in L_{p,\varphi} (\mathbf{R}_{ +})$, setting

$$ H_{2}(y): = \int_{y}^{\infty } \frac{ ( \min \{ x,y \} ) ^{\alpha } \vert \ln ( x / y ) \vert ^{\beta }}{ (\max \{ x,y \} ) ^{\lambda + \alpha }} f(x)\,dx \quad (y \in \mathbf{R}_{ +}), $$

by (30), we have

41 $$ \Vert H_{2} \Vert _{p,\phi^{1 - p}} = \biggl[ \int_{0}^{\infty } \phi^{1 - p}(y)H_{2}^{p}(y) \,dy \biggr] ^{\frac{1}{p}} < M_{2}\Vert f \Vert _{p,\varphi} < \infty . $$

### Definition 4

Define a Hardy-type integral operator of the second kind with the homogeneous kernel $T_{2}^{(2)}:L_{p,\varphi} (\mathbf{R}_{ +}) \to L_{p,\phi^{1 - p}}(\mathbf{R}_{ +})$ as follows: For any $f \in L_{p,\varphi} (\mathbf{R}_{ +})$, there exists a unique representation $T_{2}^{(2)}f = H_{2} \in L_{p, \phi^{1 - p}}(\mathbf{R}_{ +})$, satisfying for any $y \in \mathbf{R}_{ +}$, $T_{2}^{(2)}f(y) = H_{2}(y)$.

In view of (41), it follows that $\Vert T_{2}^{(2)}f \Vert _{p, \phi^{1 - p}} = \Vert H_{2} \Vert _{p,\phi^{1 - p}} \le M _{2}\Vert f \Vert _{p,\varphi}$, and then the operator $T_{2}^{(2)}$ is bounded satisfying

$$ \bigl\Vert T_{2}^{(2)} \bigr\Vert = \sup _{f(\ne \theta) \in L_{p,\varphi} (R_{ +} )}\frac{\Vert T_{2} ^{(2)}f \Vert _{p,\phi^{1 - p}}}{\Vert f \Vert _{p,\varphi}} \le M_{2}. $$

If we define the formal inner product of $T_{1}^{(2)}f$ and *g* as follows:

$$ \bigl( T_{2}^{(2)}f,g \bigr) : = \int_{0}^{\infty } \biggl( \int_{y} ^{\infty } \frac{ ( \min \{ x,y \} ) ^{\alpha } \vert \ln ( x / y ) \vert ^{\beta }}{ ( \max \{ x,y \} ) ^{\lambda + \alpha }} f(x)\,dx \biggr) g(y)\,dy, $$

then we can rewrite Corollary 2 (for $\mu_{1} = \mu $) as follows.

### Corollary 4

*If* $p > 1$, $\frac{1}{p} + \frac{1}{q} = 1$, $\beta > - 1$, $\mu = \lambda - \sigma > - \alpha $, *then the following conditions are equivalent*:

(i) *There exists a constant* $M_{2}$, *such that*, *for any* $f(x) \ge 0$, $f \in L_{p,\varphi} (\mathbf{R}_{ +})$, $\Vert f \Vert _{p,\varphi} > 0$, *we have the following inequality*:
  42 $$ \bigl\Vert T_{2}^{(2)}f \bigr\Vert _{p,\phi^{1 - p}} < M_{2}\Vert f \Vert _{p,\varphi}. $$
(ii) *There exists a constant* $M_{2}$, *such that*, *for any* $f(x)$, $g(y) \ge 0$, $f \in L_{p,\varphi} (\mathbf{R}_{ +})$, $g \in L_{q,\phi } (\mathbf{R}_{ +})$, $\Vert f \Vert _{p,\varphi}$, $\Vert g \Vert _{q,\phi } > 0$, *we have the following inequality*:
  43 $$ \bigl( T_{2}^{(2)}f,g \bigr) < M_{2} \Vert f \Vert _{p,\varphi} \Vert g \Vert _{q,\phi }. $$

*We still have* $\Vert T_{2}^{(2)} \Vert = k_{1}(\mu) \le M_{2}$.

### Remark 5

Theorem 4 and Corollary 4 are equivalent.

## Conclusions

In this paper, by means of real analysis and weight functions a few equivalent conditions of two kinds of Hardy-type integral inequalities with the non-homogeneous kernel and parameters are obtained by Theorem 1, 2. The constant factors related to the gamma function are proved to be the best possible. We also consider the operator expressions in Theorem 3, 4. The dependent cases of homogeneous kernel are assumed by Corollary 1-4. The method of weight functions is very important, it is the key to help us proving the main inequalities with the best possible constant factor. The lemmas provide an extensive account of this type of inequalities.
